# Anticipated Benefits and Concerns of Sharing Hospital Outpatient Visit Notes With Patients (Open Notes) in Dutch Hospitals: Mixed Methods Study

**DOI:** 10.2196/27764

**Published:** 2021-08-11

**Authors:** Sharon L Janssen, Nynke Venema-Taat, Stephanie Medlock

**Affiliations:** 1 Department of Medical Informatics, Amsterdam Public Health Research Institute Amsterdam UMC University of Amsterdam Amsterdam Netherlands; 2 EvA Servicecentrum Amsterdam UMC University of Amsterdam Amsterdam Netherlands

**Keywords:** patient portal, access to information, barriers and facilitators, survey, qualitative, open notes, mixed methods, electronic health record

## Abstract

**Background:**

The past few years have seen an increase in interest in sharing visit notes with patients. Sharing visit notes with patients is also known as “open notes.” Shared notes are seen as beneficial for patient empowerment and communication, but concerns have also been raised about potential negative effects. Understanding barriers is essential to successful organizational change, but most published studies on the topic come from countries where shared notes are incentivized or legally required.

**Objective:**

We aim to gather opinions about sharing outpatient clinic visit notes from patients and hospital physicians in the Netherlands, where there is currently no policy or incentive plan for shared visit notes.

**Methods:**

This multimethodological study was conducted in an academic and a nonacademic hospital in the Netherlands. We conducted a survey of patients and doctors in March-April 2019. In addition to the survey, we conducted think-aloud interviews to gather more insight into the reasons behind participants’ answers. We surveyed 350 physicians and 99 patients, and think-aloud interviews were conducted with an additional 13 physicians and 6 patients.

**Results:**

Most patients (81/98, 77%) were interested in viewing their visit notes, whereas most physicians (262/345, 75.9%) were opposed to allowing patients to view their visit notes. Most patients (54/90, 60%) expected the notes to be written in layman’s terms, but most physicians (193/321, 60.1%) did not want to change their writing style to make it more understandable for patients. Doctors raised concerns that reading the note would make patients feel confused and anxious, that the patient would not understand the note, and that shared notes would result in more documentation time or losing a way to communicate with colleagues. Interviews also revealed concerns about documenting sensitive topics such as suspected abuse and unlikely but worrisome differential diagnoses. Physicians also raised concerns that documenting worrisome thoughts elsewhere in the record would result in fragmentation of the patient record. Patients were uncertain if they would understand the notes (46/90, 51%) and, in interviews, raised questions about security and privacy. Physicians did anticipate some benefits, such as the patients remembering the visit better, shared decision-making, and keeping patients informed, but 24% (84/350) indicated that they saw no benefit. Patients anticipated that they would remember the visit better, feel more in control, and better understand their health.

**Conclusions:**

Dutch patients are interested in shared visit notes, but physicians have many concerns that should be addressed if shared notes are pursued. Physicians’ concerns should be addressed before shared notes are implemented. In hospitals where shared notes are implemented, the effects should be monitored (objectively, if possible) to determine whether the concerns raised by our participants have actualized into problems and whether the anticipated benefits are being realized.

## Introduction

### Background

One of the defining changes in patient care in the past few decades is the rise of the concept of patient empowerment. The World Health Organization defines empowerment as “a process through which people gain greater control over decisions and actions affecting their health” [1]. The operationalization of empowerment takes many forms, including interventions to improve patients’ knowledge and health literacy, applications and devices for better self-management, and advocating shared decision-making. An important aspect of empowerment is greater transparency in the health care process. This viewpoint is reflected in statements such as the National Academy of Medicine 2001 recommendation that “patients should have unfettered access to their own medical information” [2], and in Dutch law, which requires that patients be given a copy of their record upon request, and that electronic access should now be offered [3]. These directives have been interpreted in various ways. Many hospitals and clinics worldwide now offer patients web-based access to information such as current medications and laboratory results. However, a more controversial question is whether access to the medical record should include access to doctors’ free-text visit notes. Free-text visit notes are notes that a clinician writes about a patient’s visit in the patient record, as opposed to structured information such as lab values. Access to visit notes (and not just structured data such as lab results) is viewed as part of a movement toward greater transparency in health care [4].

The content of visit notes varies, but typically, visit notes contain the doctor’s observations, assessment (including differential diagnoses), and plan for treatment or further diagnostics. In 2010, Beth Israel Deaconess Medical Center captained a study of “open notes,” wherein 114 primary care providers experimented with shared notes by giving patients full access to their visit notes [4]. The results of this experiment were positive, with patients reporting positive effects and experiences, and clinicians reporting minimal disruption to their work [5], despite initial concerns about negative effects on documentation and taking too much time from clinicians and staff [4]. Since then, a number of health care institutions worldwide have adopted shared notes. These studies also report benefits from shared notes, such as patients feeling better prepared for clinic visits [6,7], feeling more in control of their health [7,8], and feeling that they better recall the doctor’s instructions [7,8]. However, some concerns emerged as well: patients reported difficulty understanding the notes [7], patients were offended by some content in the notes [6,9], or clinicians reported omitting information from the notes out of concern that it might offend the patient [8].

Most studies are based in the United States [6-10] and a few from Sweden [11]. In 2015, the United States entered stage 3 of Meaningful Use, which is a federal program that encourages the use of health information technology. Stage 3 requires the adoption of shared notes to receive financial incentives. Similarly, access to clinic notes is considered a right in Sweden, and most regions in Sweden have implemented shared notes in some form [12]. These incentives may lead to a more positive view of shared notes. The concerns and benefits may be different in other countries due to differences in culture, health care systems, or other differences. In the Netherlands, a patient must be given a copy of his or her full record upon request, but there is no requirement for the visit notes to be made available on the web. Hospitals in the Netherlands must have a patient portal available, so the visit note could be made available via the portal. A pilot study of shared notes has been announced, but the results have not yet been published [13].

### Objectives

Little is known about Dutch patients’ interest in shared notes, the benefits anticipated by physicians or patients, or their concerns. It is important to understand these barriers before attempting an implementation and to ensure that patients and physicians have realistic expectations of the benefits. Adding the Dutch perspective can help broaden the understanding currently reflected in the literature. Therefore, our aim is to assess the attitudes of patients and physicians regarding shared outpatient visit notes in a Dutch hospital setting and elucidate their anticipated benefits and concerns by means of a survey and interviews.

## Methods

### Overview

We chose a multimethodological (mixed methods) approach, using a short quantitative survey to gather opinions from a large number of patients and practitioners and think-aloud interviews to confirm participants’ interpretation of the survey questions and to understand the nuances behind the responses. As sharing notes with inpatients during hospitalization poses additional technical and practical challenges, we focused on shared visit notes in the outpatient setting. Technical challenges include providing equipment to view notes; practical challenges include determining the appropriate delay before releasing notes, providing bedside technical support services, ensuring privacy, and other challenges. The content of inpatient notes also differs from that of outpatient clinic notes. Both benefits and concerns are expected to differ between inpatient and outpatient settings.

### Survey

#### Development

The surveys were developed using published surveys on the topic [14-16] as a starting point. One survey contained questions about potential benefits and concerns [14], whereas the other two investigated only benefits [15,16]. Relevant questions from these studies were identified by one researcher (SLJ) and confirmed by a second researcher (SM). These questions were adapted, and new questions were added by the researchers based on the aims of our study (concerns, benefits, and attitudes toward shared visit notes). The survey was iteratively discussed and revised by the research team until all team members were satisfied with the questions.

#### Pilot Testing

The patient survey was piloted with health communication experts. The physician survey was piloted with doctors who were familiar with the procedures in the participating hospitals and the electronic patient record but were not eligible as subjects for the survey (ie, not currently practicing in the participating hospitals).

The feedback from the pilot tests were used to make the final survey for the patients (29 questions over 6 pages) and physicians (23 questions over 4 pages).

#### Participant Selection for Surveys

##### Physicians

The physician survey was sent to doctors from both an academic hospital (the same hospital where the patient survey was conducted) and a nonacademic hospital in the same region. Both hospitals use the same electronic health record system. An email was sent to all heads of all outpatient departments at the academic hospital and to a contact person at the nonacademic hospital who was asked to distribute it to the heads of departments there. The heads of the departments were asked to forward the email to all physicians working in their outpatient department. The email contained a short description of the study and the link to the survey. In addition, before sending the email, the study was introduced at a meeting of the heads of the outpatient departments at the academic hospital. The physicians’ survey was deployed on the web, using a custom form written in the PHP (PHP: Hypertext Preprocessor) programming language, and made available for 5 weeks (March 25, 2019, to May 1, 2019). No reminders were sent. Survey responses from the think-aloud interviews (described below) were added to the survey results by hand.

##### Patients

The patient survey was distributed in the outpatient clinic of a large academic hospital in the Netherlands. Adult, Dutch-speaking patients who attended the outpatient clinic were invited in person by a researcher (SLJ) to participate in the survey. Arrangements for the researcher to attend the outpatient clinic were made with the team leaders of the outpatient clinic, who are responsible for personnel in the outpatient clinic. From March 27, 2019, till April 16, 2019, the researcher went to various outpatient departments to hand out the surveys to patients. The researcher invited consecutive patients arriving in the waiting area on the days that she attended. We selected departments to include both older and younger patients, patients with chronic and acute disease, and varying seriousness of their diagnoses. The researcher (SLJ) approached the patient in the waiting rooms and introduced herself, the study, and the duration of the survey (5-10 minutes). If the patient agreed to participate, the researcher handed out the survey on paper and left the patient to fill in the survey. The same method was used to recruit patients for interviews; the first patients from each waiting area who were approached for the survey were asked to complete the survey with the researcher present in a think-aloud interview. Data on patients who declined to participate was not collected. Data from the paper surveys were transcribed to a spreadsheet by one researcher (SLJ).

#### Aggregation and Coding of Data

The data analysis consisted of simple counts and percentages. Patients and physicians were allowed to skip questions; therefore, we analyzed each question with n equal to the number of responses to that question. Responses to open questions were coded by one researcher (SLJ) using open coding (manifest content analysis), and the codes were discussed with a second researcher (SM).

The introductory text of both the patient and physician surveys informed participants about the study and that all data would be stored and processed anonymously. Both surveys were voluntary, and no incentives were offered to the patients or physicians.

### Think-Aloud Interviews

#### Overview

Following the methods of Westerman et al [17], we asked a subsample of physicians and patients to fill in the survey, and “think aloud” about their reasoning while filling in the answers. The researcher asked prompting questions if the respondent did not explain their answer out loud. All think-aloud interviews were performed by the first author (SLJ), a master’s student in medical informatics. This researcher’s experience with surveys and qualitative research included courses and an internship during her bachelor’s and master’s degrees. The patients and physicians were informed of the name and background of the first author and were informed of the reason for this study and asked to participate anonymously. The researcher had no relationship with the patients or physicians before the study period. All interviews were audio-recorded and transcribed by the first author.

#### Participant Selection for Think-Aloud Interviews

##### Physicians

In the email used to invite physicians to participate in the survey at the academic hospital, doctors were also invited to contact the researchers to participate in an interview. Interviews were continued until saturation was reached in the responses, and all physicians who responded were interviewed.

##### Patients

As part of the process of distributing the surveys, if an extra exam room was available in the outpatient clinic, the researcher invited patients to take the survey in the room and “think aloud” while completing it. We used a purposive sampling method: 1 or 2 patients were invited to be interviewed in each department visited while distributing the surveys until saturation was reached in the interview results.

#### Aggregation and Coding of Data

The transcribed recordings were coded by a single researcher (SLJ) using thematic analysis. A predetermined starting set of codes was used based on the constructs underlying the survey questions. Open coding was used to classify items that did not fit in the predetermined set. Two coded interviews were checked by a second researcher (SM).

### Ethics

The Medical Ethics Committee issued a waiver for this study, indicating that it does not fall under the Human Research Law of the Netherlands and that no further ethical approval is needed. Informed consent was obtained from all subjects.

## Results

The survey instruments are given in Multimedia Appendix 1 [14-16] and annotated with the references used in developing the surveys. The original surveys were in Dutch; they were translated to English by a native English speaker (SM).

### Survey

#### Physician Survey

##### Participants

A total of 350 physicians completed all (321/350, 91.7%) or a part (29/350, 8.2%) of the survey. An additional 15 empty responses (where the survey was viewed but no questions were answered) were excluded. The demographics of the participants are presented in Table 1. For physicians who were interviewed who had not completed the survey at the time of the interview, their responses to the survey during the interview were counted as part of the survey responses (8/350, 2.2%). Of the two participating hospitals, 72.8% (255/350) were from the academic hospital, and 17.4% (61/350) were from the nonacademic hospital (the remaining 34/350, 9.7% of respondents skipped this item).

**Table 1:** Demographic characteristics of the physicians (N=350)^a^.

Characteristics			Responses, n (%)
**Gender (n=314^b^)**			
	Female	174 (55.4)	
	Male	138 (43.9)	
	Other	2 (0.6)	
**Age (years; n=314)**			
	18-28	6 (1.9)	
	29-39	117 (37.2)	
	40-49	100 (31.8)	
	50-59	72 (22.9)	
	60-69	19 (6.1)	
**Location (n=316)**			
	Academic hospital	255 (80.6)	
	Nonacademic hospital	61 (19.3)	
**Department (n=320)**			
	Other (free text)	91 (28.4)	
	Gynecology, obstetrics, and gender	27 (8.4)	
	Hematology	25 (7.8)	
	Lung department	24 (7.5)	
	Internal medicine	23 (7.1)	
	Cardiology	21 (6.5)	
	Surgery	18 (5.6)	
	Departments with <5% of participants each (12 departments)	67 (20.9)	

^a^N indicates the total number of participants who filled in any demographic information.

^b^The n for each question indicate the number of participants who answered that specific question.

Respondents who selected “other department” could fill in a free textbox; the most common department given in this group (11/320, 3.4%) was the pediatric medicine department.

##### Opinions on Sharing Notes: Physician Survey

Most physicians in this survey would prefer not to share the visit notes with patients (282/345, 81.7%). When asked what information is in the visit notes, physicians indicated that their visit notes contain the anamnesis (343/350, 98%), treatment plan (339/350, 96.8%), diagnosis (328/350, 93.7%), physical examination (325/350, 92.8%), interpretation and/or summary (321/350, 91.7%), differential diagnosis (315/350, 90.0%), laboratory results (314/350, 89.7%), additional examinations (315/350, 90%), and medical history (283/350, 80.8%). The subjects that physicians were most concerned about sharing with patients were the differential diagnosis (196/350, 56%), interpretation and/or summary (162/350, 46.2%), and anamnesis (110/350, 31.4%).

Reasons why the physicians would not like to share the notes or a part of the note with the patients are given in Table 2. Participants were able to select any number of responses.

**Table 2:** Reasons why physicians do not want to share (part of) the visit note (N=350)^a^.

Reason	Responses^b^, n (%)
It will make the patient confused and anxious	236 (67.4)
The patient will not understand the content of the note	221 (63.1)
Not all of the information is relevant for the patient	205 (58.5)
I have not (yet) spoken with the patient about all the information in the note	201 (57.4)
Some information in the note is only relevant for colleagues	165 (47.1)
I expect many more questions from the patient	154 (44)
It will generate more work	122 (34.8)
The visit note is my personal note	113 (32.2)

^a^The total “N” indicates the total number of physicians who responded to this question.

^b^Number of participants choosing this response.

We also allowed respondents to fill in free-text reasons why they did not want to share (part of) the visit notes, which were added by 20% (70/350) of respondents. Analysis of these free-text comments underscored concerns about confusing and worrying the patient, especially by reading the differential diagnosis. Doctors pointed out that they often need to consider the possibility of an unlikely but worrisome diagnosis such as cancer or amyotrophic lateral sclerosis (Lou Gehrig disease) and may want to watch for signs of it on diagnostic tests but do not want to discuss it with the patient unless there is a substantial chance that the patient actually has this disease. Doctors also remarked that the notes are a place to record their thoughts so they can pick up their train of thought later on and that these thoughts might not be complete or may later be proven wrong. This is especially problematic when the doctor suspects a sensitive problem, such as abuse or sexually transmitted diseases. The record also functions as a tool to facilitate discussion of these matters with colleagues. Doctors also expressed concern about family members reading the file or that information would be left out or displaced to other parts of the record, thus compromising the quality of care.

Physicians were largely unwilling to write their notes with less jargon and abbreviations (193/321, 60.1%) to make it more understandable for patients. A smaller group of physicians were willing to partly change their writing style (85/321, 26.4%), and the rest were willing to change their writing style (43/321, 13.3%).

##### Benefits and Concerns: Physician Survey

In addition to asking physicians about their reasons for sharing or not sharing their visit notes, we also asked physicians about the anticipated benefits of sharing notes with patients and about their concerns. Although there is some overlap with reasons for not sharing notes, we asked about them separately as a doctor may have a concern but not consider it a reason to avoid sharing notes. Participants were able to select any number of responses. The results are presented in Tables 3 and 4.

**Table 3:** Anticipated benefits according to physicians (N=322)^a^.

Potential benefit	Responses^b^, n (%)
The patient can better remember and understand what was said during the visit	144 (44.7)
It facilitates shared decision making	98 (30.4)
The patient is better informed about their illness and health	96 (29.8)
The patient can make corrections or additions	82 (25.4)
The patient can put the already-accessible results^c^ in context	47 (14.5)
The family of the patient will be better informed	46 (14.2)
It facilitates a better doctor-patient relationship	37 (11.4)
Patient compliance will increase	23 (7.1)

^a^The total “N” indicates the total number of physicians who responded to this question.

^b^Number of participants choosing this response.

^c^Patients already have access to laboratory results via the patient portal.

**Table 4:** Anticipated concerns according to physicians (N=322^a^).

Potential concern	Responses^b^, n (%)
The patient will be confused because they won’t understand the content	276 (85.7)
The patient will be worried (eg, by the differential diagnosis)	276 (85.7)
I will need to answer more questions via the portal	239 (74.2)
The patient will ask for corrections and/or additions	215 (66.7)
I will have difficulty communicating with my colleagues because some of the information would be perceived differently by the patient	203 (63)
It will generate more work	200 (62.1)
I will need to spend more time on documentation	200 (62.1)
The family of the patient will interfere	130 (40.3)
I will need to explain more to the patient during the consult	117 (36.3)

^a^The total “N” indicates the total number of physicians who responded to this question.

^b^Number of participants choosing this response.

We also offered a free textbox where physicians could fill in other benefits or concerns, which was filled by 28.8% (93/322) of the participants. Analysis of the free-text responses showed that 23.9% (77/322) of physicians said that they believed that there are no benefits for the patients or physicians. Other comments (16/322, 4.9%) underscored the benefits of increased retention of information, self-efficacy, and the importance of transparency, as well as the concern that patients will not understand what is written.

#### Patient Survey

##### Participants

A total of 99 patients participated in the survey. The demographics of the respondents are presented in Table 5.

**Table 5:** Demographic characteristics of patients (N=99)^a^.

Characteristics			Responses, n (%)
**Gender (n=94)^b^**			
	Female	68 (72)	
	Male	26 (28)	
**Age (years; n=94)**			
	<18	1 (1)	
	18-28	17 (18)	
	29-39	15 (16)	
	40-49	10 (11)	
	50-59	22 (23)	
	60-69	20 (21)	
	70-79	9 (10)	
**Highest education level (n=83)**			
	Primary school	1 (1)	
	High school	39 (46)	
	University	43 (52)	
**Self-reported health (n=88)**			
	Very poor	2 (2)	
	Poor	20 (23)	
	Fine	51 (58)	
	Very well	13 (15)	
	Excellent	2 (2)	

^a^The total “N” indicates the number of participants who filled in any demographic information.

^b^The “n” value for each question indicates the number of participants who answered that specific question.

##### Opinions on Sharing Notes: Patient Survey

In contrast to the physicians, most patients found it important (50/90, 56%) or very important (19/90, 21%) to read the visit notes in the patient portal. Patients were most interested in seeing their laboratory results (78/89, 88%), a summary of the visit (71/89, 80%), and the diagnosis or differential diagnosis (60/89, 67%). Other parts that interested patients were the treatment plan (45/89, 51%), medication (41/89, 46%), and physical examination (34/89, 38%).

Patients agreed (27/90, 30%) or strongly agreed (28/90, 31%) that they expected notes to be written in understandable language or layman’s terms if the doctors know that they will be reading the notes. Patients indicated that if they did not understand the notes, they would ask the doctor at the next visit (39/90, 43%), discuss with family or friends (16/90, 18%), call the department (12/90, 13%), or send a message to the doctor (10/90, 11%). We also asked the patients what they would do if they were to see information in the note that they disagreed with or did not expect. Patients indicated that they would ask the doctor about it during the next visit (62/90, 69%), call the department (38/90, 42%), or send a message to the doctor (22/90, 24%).

Patients were also asked about what they would do with the information in the notes, the benefits they anticipate from open notes, and their concerns (Table 6).

**Table 6:** Responses to statements on anticipated responses to notes, possible benefits, and concerns about shared notes.

Statement	Strongly disagree, n (%)	Disagree, n (%)	Neutral, n (%)	Agree, n (%)	Strongly agree, n (%)
Discuss the notes with my doctor or another doctor (n=89^a^)	2 (2)	12 (14)	25 (28)	39 (44)	11 (12)
Discuss the notes with family and friends (n=90)	8 (9)	16 (18)	27 (30)	32 (35)	7 (8)
I would better remember what was discussed at the visit (n=90)	3 (3)	2 (2)	14 (16)	50 (56)	21 (23)
I would feel more in control of the care process (n=90)	4 (4)	5 (6)	26 (29)	40 (44)	15 (17)
I would better understand my illness and health (n=90)	5 (5)	23 (26)	30 (33)	42 (47)	12 (13)
The notes will be more confusing than useful (n=90)	8 (9)	36 (40)	32 (35)	8 (9)	6 (7)
I would worry more about my health (n=89)	9 (10)	42 (47)	29 (33)	6 (7)	3 (3)

^a^The “n” value for each statement indicates the number of participants who responded to that specific question.

### Think-Aloud Interviews

#### Physician Interviews

##### Participants

In total, 13 physicians were interviewed. The duration of the physician interviews was 20-25 minutes. The interview participants were 38% (5/13) male and 62% (8/13) female and worked in 10 different departments.

##### Reasons for Not Wanting to Share Visit Notes: Physician Interviews

Physicians commented on their reasons for not wanting to share the visit notes with patients (Table 7). The most common reason was fear that the information would be confusing or misleading for the patient:

As a dermatologist we know 3000 skin diseases. So for each spot, an experienced doctor could think of 10 to 20 diagnoses. This would be very confusing for the patient.

**Table 7:** Coded reasons for not sharing visit notes (n=13).

Reason	Participants, n (%)^a^
Will be confusing or patient would not understand	7 (54)
Not relevant for patient	6 (46)
Insecurities with differential diagnosis	5 (38)
Sensitive data, nuances, or psychological and social information	4 (31)
Considerations	4 (31)
No possibility of a note with colleagues	4 (31)
Did not discuss everything	3 (23)
Discuss when I am 100% sure	3 (23)
File for the doctor	3 (23)
No benefit for patient health care	3 (23)

^a^Number of participants who mentioned a reason classified under this code.

Physicians also mentioned the need to document sensitive data, including things such as the suspicion of domestic violence:

Now and then you are suspecting domestic violence or something else. Before you will mention it to the authorities, you need to be sure and gather some evidence.

Physicians also mentioned the function of the patient record as a cognitive aid for the diagnostic process and the need to note clinical hunches that may not yet be confirmed:

I don’t have any secrets from my patients, never. So, from that point of view they are allowed to read the notes - but the point is that I need a space for considerations, worries, and fears. You need to write that somewhere.

##### Benefits and Concerns: Physician Interviews

In addition to the concerns in the structured part of the survey, doctors mentioned two additional concerns. First, notes are sometimes entered by doctors in training, and they may not yet have the skills to communicate in a way that is appropriate for patients. Physicians also mentioned the need to document private conversations with minors and whether their parents would have access to the notes:

One of my patients just turned 18 and she has a worrying family situation. Her dad was very controlling and I think that they will make comments about my interpretation that it is not only her [that has a problem], because her diagnosis is supported by factors such as stressful family situation.

##### Jargon: Physician Interviews

We asked the physicians if they were willing to write the notes in understandable language for the patients, and 6 of them said no. Four physicians mentioned that jargon is the most efficient way to work for them: “Every higher education, where intellectual effort is needed and everything that has to do with professionalism has jargon.”

##### Differences in Visit Note Between Departments: Physician Interviews

In total, 3 physicians said that there is a difference between the notes of different departments. The example was given that notes about a fractured hip (orthopedics) are very different from the notes from hematology.

##### Other Ways to Achieve the Benefits: Physician Interviews

Half of the physicians wanted to have more time and communication with the patient; 3 physicians mentioned that they were writing information on paper during the visit. Physicians also mentioned sharing letters (sent to the general practitioner) or sharing the visit summary as better alternatives.

#### Patient Interviews

##### Participants

In total, 6 patients were interviewed. The average duration of the patient interviews was 10-15 minutes. Some patients asked if their partner could attend the interview, so in two interviews, nonparticipants were present. Participants were 50% (3/6) female and 50% (3/6) male, falling into five different age categories (ranging from 18-28 years to 70-79 years).

##### Reasons for Wanting Open Notes: Patient Interviews

All patients who participated in the interviews were interested in seeing the visit notes but named various reasons for being interested (Table 8). The most cited reason was curiosity; the second was feeling that the note was about them, and this itself is a reason to see it: “I would like seeing the notes because it is about me and I think it is very important that I know exactly what happened.” Patients also mentioned that seeing the notes would help them remember what was discussed, especially after receiving bad news from the physician: “Nine out of ten times you will hear the half of what the doctor is saying.” All 6 patients said that they were interested in seeing all parts of the note.

**Table 8:** Coded reasons for wanting to access visit notes (n=6).

Reason	Participant, n (%)^a^
Curious	4 (67)
It’s about me	3 (50)
Medical background	3 (50)
Cannot remember everything	2 (33)
Bad news	2 (33)

^a^Number of participants who mentioned a reason classified under this code.

##### Use of Medical Jargon: Patient Interviews

Patients also had varied opinions about expecting a note to be in layman’s terms (Table 9); 2 of the patients interviewed believed that the note should be in layman’s terms:

If the note contains a lot of abbreviations than it makes no sense for the patient to read the note because I would not understand half of the note.

However, 2 patients were neutral on the topic, and 2 disagreed:

No, I don’t expect it because the doctors need to have the possibility to talk and speak in their jargon.

**Table 9:** Comments on the use of medical jargon (n=6).

Comment	Participants, n (%)^a^
Doctors need jargon	3 (50)
I would not understand the notes	3 (50)
Already administrative burden	3 (50)
Works easier for doctors	2 (33)
Prefer it but do not expect it	1 (17)

^a^Number of participants who mentioned a reason classified under this code.

##### Benefits and Concerns: Patient Interviews

In total, 3 patients mentioned privacy as a potential concern with open notes, both in terms of internet security and who might be given access to the notes in addition to the patient themselves (eg, family members or home care workers).

##### Other Ways to Achieve the Benefits: Patient Interviews

Patients suggested that some of these benefits could be achieved by bringing someone with you to the visit and having good informational leaflets.

## Discussion

### Principal Findings

We designed and conducted surveys regarding opinions on shared notes from 350 physicians and 99 patients, and conducted interviews with 13 doctors and 6 patients. Of all participants, 81.7% (282/345) of doctors prefer not to share visit notes with patients. Physicians indicated that nearly all aspects of care appear in the note, and they were particularly concerned about patients reading the differential diagnosis, the interpretation or summary, and the anamnesis. The most common reasons were worries that reading the note would make patients confused and anxious, that the patient will not understand their notes, that the information is not relevant for the patient, and that the note may contain information that has not yet been discussed with the patient. Clinical notes are written using medical jargon, and most physicians (193/321, 60.1%) did not want to change their writing style to make it more understandable for patients. Physicians did anticipate some benefits, such as better patient recall of what was discussed, better shared decision-making, and keeping patients informed. However, 23.9% (77/322) indicated that they saw no benefit in allowing patients to access the visit notes. Physicians also had many concerns (with some overlap with their reasons for not wanting to share notes), including unnecessary confusion and worry for the patient and family, needing more time to answer patient questions and more time for documentation, and more difficulty communicating with colleagues via the notes. The interviews clarified that physicians were concerned about the need to document sensitive information, such as the suspicion of domestic violence, and the need to have a place to document conversations with minors. They also mentioned the function of the patient record as a cognitive aid to sort through unconfirmed thoughts. Physicians were also concerned about patients reading notes written by doctors in training, who might not write things in a way that is appropriate for the patients. In the patient survey, 77% (69/90) of patients found it important or very important to see their visit notes. Patients were most interested in viewing their laboratory results, visit summary, and diagnoses. Most patients (55/90, 60%) expected visit notes to be written in layman’s terms. Most patients indicated that if they had questions, they would ask them at the next visit, although some (12/90, 13%) indicated that they would call the hospital to ask. A higher percentage indicated that they would call (38/90, 42%) or send a note (22/90, 24%) if they found information that they did not agree with or did not expect. Patients saw some potential benefit to reading their notes: they felt they would better remember what was discussed, feel more in control, and better understand their health. Generally, patients did not feel they would worry more, and 49% (44/90) felt they would not find the notes too confusing (although 32/90, 35% were not sure, and 14/90, 16% felt they would find the notes confusing). The patients who were interviewed were mainly interested in seeing the notes out of curiosity and because they felt they have the right to see information that is written about them. Patients also noted that it is difficult to remember everything from the visit, especially after receiving bad news, and reading the notes would help. The patients interviewed also mentioned security and privacy concerns with shared notes.

### Strengths and Limitations

A major strength of this study is the use of mixed methods to gather opinions from both physicians and patients. The survey allowed us to gather opinions from a broad sample of physicians and patients, whereas the interviews allowed us to gain insight into the thoughts behind the responses. This gives us a good picture of the current mindset of these two major stakeholder groups. Another strength is the broad sample of participants, with physicians from both an academic and a nonacademic hospital and a variety of departments. However, this study had some important limitations. The survey that we used was not validated; to our knowledge, no validated survey exists on this subject. We created a survey based on the literature and pilot tested it before deploying it, ensuring that the survey questions were clear and complete according to our pilot participants. We cannot determine the response rate because we do not know how many physicians were invited or how many read the invitation email. To ensure anonymity, we did not attempt to prevent the same person from filling in the survey multiple times, although we saw no evidence of this. We also did not document any information about patients who declined to participate in the survey. The age and gender of the physicians who responded were approximately similar to the demographics of physicians in the participating hospitals. The patients who participated were more likely to be female, which might be due to a higher percentage of women in some clinics (eg, gynecology), a general participation bias (as women are generally more likely to participate in surveys or studies [18]) or may be due to a participation bias in patients who perceived themselves as similar to the researcher who distributed the surveys (who is also female). We did not ask patients about their medical conditions, and waiting rooms were shared between several outpatient clinics. Thus, our method should provide some variety in the medical conditions of patient participants, but we do not know how much. Physicians and patients with strong feelings about shared notes may be more likely to participate. The fact that all the doctors who were interviewed had a predominantly negative impression of shared notes suggests a participation bias in the interviews. Another potential source of bias is that one researcher invited patients to the surveys, entered data from paper surveys, conducted the interviews, and performed the transcribing and coding. However, a sample of the interviews and coding was checked by a second researcher to reduce the risk of bias. No field notes were made during the interviews, and the transcripts were not checked by the participants. Finally, the choice of hospitals was based on convenience, and therefore, the responses might not be representative of all Dutch hospitals. However, we included physicians from 2 centers, one academic and one nonacademic, and succeeded in including participants from a broad sample of departments.

### Comparison With Previous Work

Two previous studies have investigated clinicians’ opinions before the implementation of shared notes; both were focused on psychiatric care, one in the United States [10] and one in Sweden [11]. In contrast to our study, 82% of participants in the US study were positive about shared notes [10]. The Swedish study did not explicitly ask participants if they wanted to share their notes [11]. Participants in both of the aforementioned studies expressed concerns similar to those in our study: causing unnecessary worry for the patient (77% and 58%, respectively), being more confusing than helpful (67% and 53%, respectively), spending more time answering questions outside of visits (46% [10]) or being contacted with questions (69% [11]), and details being omitted from the notes (69% [10]) or being less candid in the documentation (42% [11]). In addition to the issues raised in previous studies, our physicians also expressed concern that additional time needed for documentation.

One previous study gathered patient opinions before implementation in ophthalmology patients in the United States [19]. Similar to our patients, those in this earlier study were positive about shared notes (95%). Patients felt that it would help them to better: understand their conditions (95%), remember their care plan (94%), feel more in control (90%), be prepared for visits (89%), and take better care of themselves (84%). Unlike our patients, patients in this study also believed it would help them to take their medications (77%) and rated their own anticipated ability to understand the notes as 7.5 out of 10. Studies conducted after the implementation of shared notes have found that the perceived benefits and concerns were similar to those found before implementation in both clinicians [20-23] and patients [6,7,9,12,24-27]. However, all outcome measures in these studies were assessed subjectively, with the exception of Ross and Lin [21], who found that the number of messages from the patient to the doctor increased by 31% after the implementation of shared notes. Thus, for the most part, we still do not know if the concerns raised in our study are likely to manifest or if the perceived benefits will be realized if shared notes are implemented.

### Interpretation and Implications

One important finding in our study is that many patients expect the note to be written in layman’s terms, whereas many physicians do not want to change the way their notes are written to make them more understandable to patients. This mismatch of expectations must be addressed if the benefits of shared notes are to be realized—patients must understand the notes in order for them to have any benefit. However, clinical jargon exists because it is a precise and efficient language for physicians to document findings and communicate with colleagues. Physicians are rightfully concerned that having to include a plain-language explanation of jargon terms with every clinical note would increase documentation time, ultimately adversely affecting patient care. However, a possible solution to this could be the automated interpretation of clinical notes. van Mens et al [28] have reported promising results in their efforts to translate diagnoses to layman’s terms using SNOMED-CT (Systematized Nomenclature of Medicine-Clinical Terms); similar technology could be used to produce an explanation in layman’s terms while still allowing physicians to communicate effectively with one another.

Another major concern raised by physicians is the need to document sensitive information. This is supported by Erlingsdóttir et al [29], who also reported concerns about patient privacy and confidentiality in their analysis of 1554 free-text answers from two web surveys conducted among health care providers in Sweden. Examples raised by our participants included the need to document communication with a minor in situations where the parents have mental health issues, the need to document cases of suspected abuse, and the need to document problems that the patient themselves has not yet accepted. Physicians were also concerned about how patients would respond to reading the differential diagnoses, which often contain some worrisome possibilities. Physicians feared that this important information would either be documented in other parts of the record, making it more likely to be missed on subsequent visits, or simply not be documented at all, which poses serious risks for patient care.

Another potential issue raised by our participants was the notes written by trainees. This is supported by Kung et al [30], who found that 20% of notes written by trainees raised some concerns. Trainees may be more likely to inadvertently use language that is offensive to patients. As part of the learning process, trainees must create a differential diagnosis list. The differential is the part of the notes that our physicians were most worried about sharing, as it often contains at least some alarming (although usually unlikely) possibilities. A possible solution is to document the differential and trainee notes in another part of the record; however, this runs the risk of fragmenting the record and making information more difficult to find. Another risk is that the visit note effectively becomes a note only for patients, with only a cursory summary, and that the “real” notes simply move to another field in the record.

For the most part, the findings from our patient survey and interviews were in line with previous research. In addition to the questions drawn from previous surveys, we asked our participants what they would do if reading the notes raised questions. They indicated that they would most likely search on the internet or ask at the next appointment; only a minority indicated that they would call the clinic or send a message via the patient portal. This may indicate that the increase in workload resulting from sharing visit notes would be manageable. Our patients also raised concerns about security and privacy, both in the technical sense and socially (eg, whether informal caregivers also have access to the notes).

These findings are important for hospitals seeking to implement shared notes, both in the Netherlands and elsewhere. In the Netherlands, the implementation of shared notes would consist of releasing the notes to patients in the patient portal. The concerns raised by the physicians and patients in our study should be investigated and addressed before implementation is attempted. Care should be taken to sincerely address these concerns to avoid maladaptive responses, such as moving clinical documentation to other parts of the record. Particular attention should be paid to departments who have pediatric and adolescent patients, especially in situations where giving parents access to the record may lead to harm to the patient. Differences in the content of visit notes between departments should also be considered, as well as differences between patients (eg, patients with chronic diseases may understand more of the jargon about their disease than patients with acute disease). We should also take note of the benefits that patients and physicians see in sharing the notes and find a solution that best delivers these benefits while avoiding the pitfalls foreseen by our participants.

Future research should investigate these possible solutions, preferably with the measurement of objective outcomes alongside subjective outcomes. Some important outcomes are inherently subjective, such as patients’ trust in the health care system and sense of empowerment. However, the effects on communication and workflow can and should be measured objectively, such as the time needed for documentation, the ability of other physicians to find needed information, and patients’ understanding of their medical situation. Future work should also repeat some of the questions presented in our survey but with an example of a visit note, so that patients are better able to say whether they are interested in the content of the note and can understand it. Future studies could also explore the relationship between factors such as health status and interest in and perceived benefits of open notes. Patients with poor health may have less energy to read notes or may be even more interested in their notes than patients with better health.

### Conclusions

This mixed methods study investigated patients’ and physicians’ opinions of shared visit notes in the outpatient clinic setting in the Netherlands. Patients generally favored sharing notes (70/90, 77%), whereas physicians were often opposed (282/345, 81.7%). We found a mismatch between patients’ and physicians’ expectations for the language used in clinical notes; patients expected notes in layman’s terms, whereas physicians need to communicate using precise clinical terms. Physicians raised concerns about documenting sensitive information, worrying patients with clinical suspicions and the differential diagnosis, and poorer communication due to fragmenting of the clinical documentation; patients raised concerns about security and privacy. Patients and a minority of physicians saw potential benefits in providing patients with better insight into their health state and better retention of important information from the patient visit. Hospitals seeking to implement shared notes should investigate and address these concerns, and future work should measure the effects of shared notes (objectively, when possible) to better understand if the concerns manifest as problems and if the anticipated benefits are realized.

## Appendix

Multimedia Appendix 1 Physician and patient survey instruments.

## Abbreviations

PHP: PHP: Hypertext Preprocessor
SNOMED-CT: Systematized Nomenclature of Medicine-Clinical Terms
