# Supplementary material for: Anticipated Benefits and Concerns of Sharing Hospital Outpatient Visit Notes With Patients (Open Notes) in Dutch Hospitals: Mixed Methods Study
Source: J Med Internet Res. 2021 Aug 11;23(8):e27764. doi: 10.2196/27764 (PMC8387887; doi:10.2196/27764)
Supplement: Multimedia Appendix 1 [file jmir_v23i8e27764_app1.docx]

Appendix

Survey patients

**Questionnaire about visit notes in the patient portal**

During or after an appointment with a patient, doctors make a visit note / visit report. This note is made by the doctor to record what was discussed during an appointment. In general, a visit note contains a summary of the appointment, physical examination, results of possible examinations, a diagnosis / possible diagnoses to be investigated, and the management / treatment plan. Notes are often written in doctor's language, because it is faster for doctors to read and write. This means that medical terms and Latin abbreviations are used in many notes.

We are currently conducting a study about sharing a doctor's notes with the patient via the patient portal. <Name of portal> is the patient portal of <name of hospital>. You do not need to have a patient portal account to participate in this study. With the help of this questionnaire we want to map the opinions and feelings of patients. In this questionnaire, general questions about the portal are asked and we ask for your opinion and feelings regarding viewing the visit note. For some questions, an explanation is requested: you can recognize this by the addition 'why'? or 'explanation' ?. For these questions, please try to give an explanation.

Your answers to this questionnaire are stored and processed anonymously. By filling in this questionnaire, you give permission for use of this data.

Thank you in advance for completing this questionnaire!

***Patient portal account***
The patient portal of <name of hospital>

1. Do you have a patient portal account?

€ Yes

€ No      

1. **If “ yes ” to question 1**, how often have you used the patient portal in the past six months?

€ Number of times: …………………………

1. **If “ no ” to question 1 or “ 0 times ” to question 2**: why do you not have a patient portal account or why not use your patient portal account?
   **You can choose multiple answers**

€ No new information is available

€ I cannot log in (anymore)

€ I am not interested in the new information

€ I do not have a computer and internet

€ I find it too complicated to use

€ I don't need it

€ I think the patient portal is too limited

€ I am concerned about my privacy

€ Other, namely ……………………

1. **If “ 1 or more times ” in question 2 :**What do you mainly use the patient portal for?

**You can choose multiple answers**

€ View results

€ Request and view appointments and visits

€ Ask a healthcare provider a question / request a repeat prescription

€ View letters

€ Complete questionnaires

€ View health summary

€ Download the record

€ Current health problems

€ Medication, allergies, vaccinations

€ Share my data (Share Everywhere, Care Everywhere )

***Visit notes***
*The following questions are about seeing the visit notes in the patient portal that the doctor creates in the record.*

1. Would you be interested in reading your doctor's notes in the patient portal?^B^

€ Yes

€ No.

**Explanation :**

…………………………………………………………………………………………………………………………………… ….

…………………………………………………………………………………………………………………………………… ….

…………………………………………………………………………………………………………………………………… ….

1. **If you answered “ no ” to question 1.**

You do not have a patient portal account; if these visit notes from the doctor appear in the patient portal, will you create an account?

€ Yes

€ No.

€ I don't know

If you **have entered “no”**at **question 6**, you can stop the questionnaire. Thank you for completing the questionnaire and we will process and store your answers anonymously.

1. Which part of the notes would you be most interested in?

**You can choose multiple answers**

€ A summary of the consultation

€ Physical examination

€ Results of tests

€ Diagnosis / possible diagnoses

€ Policy / discussion

€ Medication

€ Other ……………………………………………

As described above, a visit note / note is often written with medical terms and Latin abbreviations. Also can the visit notes vary in usage per doctor.

1. If the doctor knows that you are also reading the notes, do you expect the note to be written in plain language for you ?

€ Strongly disagree  € Disagree €  Neutral  € Agree  € Strongly agree

1. What do you think if the note is written in doctor's language (ie medical Latin terms and abbreviations)?

…………………………………………………………………………………………………………………………………… ….

…………………………………………………………………………………………………………………………………… ….

…………………………………………………………………………………………………………………………………… ….

1. What will you do if you see difficult terms and abbreviations in the note that you don't understand?

**You can choose multiple answers**

 Search the internet yourself

€** **Discuss with family & friends

 Send messages to the doctor via the patient portal

 Call the department where the doctor works

€ Ask the doctor at a subsequent visit to the clinic

 Other, ……………………………………………….

1. What do you do if you see information in the note that you disagree with or come across information that you did not expect?

**You can choose multiple answers**

 Search the internet yourself

€ Discuss with family & friends

 Send messages to the doctor via the patient portal

 Call the department where the doctor works

€ Ask the doctor at a subsequent visit to the clinic

 Other ……………………………………………….

***Opinions about visit notes***
Below are a number of statements and open questions about the visit note that the doctor makes.

1. I think it is important to read the notes.^B^

 Not important at all   Not important    Neutral  Important   Very important

1. I will discuss the doctor's notes with my doctor, another doctor, or my general practitioner.

 Strongly disagree   Disagree   Neutral   Agree   Strongly agree

1. I will discuss the doctor's notes with family and / or friends.

 Strongly disagree   Disagree   Neutral   Agree   Strongly agree

*Possible drawbacks of reading notes*

1. The notes will be more confusing than helpful.^A^

 Strongly disagree   Disagree   Neutral   Agree   Strongly agree

1. I would be more concerned about my health after reading the note.^A^

 Strongly disagree   Disagree   Neutral   Agree   Strongly agree

1. Do you have any other concerns if the notes become visible in the patient portal?

…………………………………………………………………………………………………………………………………… ….

…………………………………………………………………………………………………………………………………… ….

…………………………………………………………………………………………………………………………………… ….

*Possible benefits of reading notes*

1. I remember better what was discussed during the appointment.^A,B^

 Strongly disagree   Disagree   Neutral   Agree   Strongly agree

1. I would feel more in control of my care process.^A,B,C^

 Strongly disagree   Disagree   Neutral   Agree   Strongly agree

1. I would understand my health and complaints better.^A,B,C^

 Strongly disagree   Disagree   Neutral   Agree   Strongly agree

1. I would be more likely to take my medications.^A^

 Strongly disagree   Disagree   Neutral   Agree   Strongly agree

1. Are there other advantages for you if the notes become visible in the patient portal?

…………………………………………………………………………………………………………………………………… ….

…………………………………………………………………………………………………………………………………… ….

…………………………………………………………………………………………………………………………………… ….

1. It is sharing the visit notes of doctors in your opinion the best way to achieve the aforementioned advantages (question 14 t / m 17) or do you have another suggestion to achieve these benefits?

…………………………………………………………………………………………………………………………………… ….

…………………………………………………………………………………………………………………………………… ….

…………………………………………………………………………………………………………………………………… ….

***Socio- demographic and medical data***

Finally, we would like to know some background information about you.

1. What is your age?

 18 - 28 years

 29-39 years

 40-49 years

 50-59 years

 60 - 69 years

 70 –79 years

 80> years

1. What is your gender?

 Man

 Woman

 Other ……………………………………….

1. What is your highest completed education level?

 Primary school

 High school

 Trade school

 College preparatory high school

 University        

1. In general, your health at this point is:^A,B,C^

 Very bad   Bad   Good  Very good  Excellent

1. Do you have a health complaint for which you had to visit the doctor in the past year?^A^

 Yes, number of times ………………………………….

 No.

1. Do you have a health complaint that requires regular examinations or appropriate treatment?^A^

 Yes,

 No, my health doesn't change much

 I don't know      

Thank you for completing this questionnaire. We will process your data anonymously during this investigation.

Survey clinicians

**Questionnaire about visit notes in the patient portal**

During a patient's visit to your clinic, you make a visit note / visit report in the electronic health record. Currently, these visit notes / visit reports are **not released to the patient in the patient portal after they have been finalized / signed.**In America, the “Open Notes Movement” is taking place and this means that more and more doctors and hospitals are going to share their notes with patients. **We are researching the opinions of doctors and patients about the phenomenon “OpenNotes”.**By means of this questionnaire we try to map the opinions and feelings of doctors at the clinic. At the end of the questionnaire there is another option for explanations and comments. Do not use the names of patients or colleagues in your answers. By completing this questionnaire, you consent to us storing and processing your answers anonymously. Thanks in advance! For other questions you can always email <email address>.

1. What do you write in the visit note?

**Choose all that apply**

Anamnesis

History

Physical examination

Additional research

Results of investigations

Diagnosis

Differential diagnosis

Discussion / summary

Policy

Medication

Other ………………………………………… ..…

1. If you had to choose, do you think the patient should be able to read these visit notes via The patient portal?^A^

 Yes

 No.

1. Which option would you go for?

**1 choice possible**

 All progress notes are automatically shared after they are “done” in The patient portal

 I can set for each visit note whether I release it in The patient portal

 I release a certain portion of the progress report to each patient

 Depending on each patient whether I release the visit note or part of it

 Progress notes are not shared with the patient

1. Are there parts that you would rather not want released to the patient?

Choose all that apply

 Anamnesis

 History

 Physical examination

 Additional research

 Results of investigations

 Diagnosis

 Differential diagnosis

 Discussion / summary

 Policy

 Medication

 Other ………………………………………

1. What would be a reason for you not to release a note or part of the note?

Choose all that apply

 The note is your property / my personal note

 Information that is only suitable for colleagues

 Not all information is relevant to the patient

 The patient does not understand the contents of the note^A^

 I have not (yet) discussed all the information in the note with the patient

 I expect a lot more questions from the patient

 It will only confuse and frighten the patient^A^

 It gives me more work

 Other, namely …… ..

In addition to the patient, the doctor also has rights with regard to the file. A doctor has ***the right to a personal work note***. The personal work note is often interpreted differently by doctors. Below is the official definition of a personal work note from the guidelines of the KNMG. Keep this guideline in mind when answering the following questions.

**KNMG guideline: guideline for dealing with medical data**

Personal work notes are temporary notes that are intended for the purpose of forming your own preliminary thoughts and that contain impressions, suspicions or questions raised by the doctor. They are kept in a safe place outside of the medical record and the physician should ensure that they are destroyed over time or included in the medical record if they are still important for the proper care of the patient. Personal work notes are not intended to be seen by anyone other than the physician. If they are shared with others, there is no longer a personal work note. In that case, the note must be included in the medical file or destroyed. The patient is not entitled to access or a copy of personal work notes.

1. Do you need a personal work note?

 Yes

 No.

 No, but I will if the progress notes are released

 Other… ..

1. Where do you make your personal work note now?

Choose all that apply

 I don't make them

 Notebook

 Email

 I don't have the option to make a personal note in the electronic health record

 Other or I do it in the electronic health record

- - Please provide further explanation:

.................................................. .................................................. ....................... ........................... .... .............................................. ..................................................

1. Do you think if the patient can look at your visit note, you would transfer information to other notes where the patient does not have access such as personal work note, other type of note or somewhere else in the file ?

 Yes

 No.

 Partly

 Other, namely ……………………………………………….

1. Suppose that the visit notes are released and the patient can (partially) view your notes. What do you think are possible benefits ?

Choose all that apply

 The patient is better informed about his illness and health^A,B,C^

 The patient can better remember and understand what was said during the consultation^A,B,C^

 The patient is better able to put the results already released into context

 The patient will request corrections / additions

 The patient's family is better informed

 Better patient compliance^A^

 It promotes shared decision making**^C^**

Better doctor-patient relationship

 Other, namely ………………………………………………….

1. Do you think there are other ways to obtain the above benefits?
   .................................................. .................................................. .................................................. .................................................. .................................................. .................................................. .................................................. ..................................................

1. Suppose that the visit notes are released and the patient can (partially) view your notes. What do you think are possible drawbacks ?

Choose all that apply

 The patient is confused with my notes because he does not understand the content^A^

 The patient will be very concerned (e.g. due to differential diagnosis)

 The patient will request corrections / additions

 The patient's family will interfere

 It gives me more work

 I have to answer more questions via the patient portal (unpaid)

 I have to spend more time on documentation

 I have to explain more to the patient during the consultation

 I lose a communication option with colleagues

 Other, namely …………… ..

1. Are you willing to write the notes in language that is easier for the patient to understand?

 Yes

 No.

 Partly

1. I already receive a lot of questions from patients who do not understand results or who have obtained entire reports from Google and therefore ask for an explanation during the consultation

 Totally disagree   Disagree   Neutral   Agree   Totally agree

1. I think I will receive more questions from patients if they can view their notes via the patient portal

 Totally disagree   Disagree   Neutral   Agree   Totally agree

1. It is an option for me to create a note that I release to the patient and make another note with information that I do not want to disclose to the patient but want colleagues to be aware of it.

 Totally disagree   Disagree   Neutral   Agree   Totally agree

1. What is your age?

 18 - 28 years

 29-39 years

 40-49 years

 50-59 years

 60 - 69 years

1. What is your gender?

 Man

 Woman

 Other, …………………………………

1. In which hospital do you work?

 Hospital 1>

 Hospital 2>

 Other, …………………………………

1. In which **outpatient department**do you work?

|  |  |
| --- | --- |
|  Cardiology |  Ear, Nose, Throat |
|  Surgery |  Lung diseases |
|  Dermatology |  GI/Liver |
|  Endocrinology |  Nephrology |
|  Hematology |  Neurology |
|  Hyperbaric Medicine |  Oncology / GI Oncology |
|  Internal medicine |  Ophthalmology |
|  Infectious diseases |  Psychiatry |
|  Immunology & Rheumatism |  Urology |
|  Clinical Genetics / Cardiac genetics |  Reproduction, gynecology / sexual health / obstetrics / gender |
|  |  Other |

1. You generally treat patients who:

 Have life-threatening illnesses

 Do not have life-threatening illnesses

 A mix of the above option

1. Your patients are generally:

Choose all that apply

 Short-term patients (1 year or less)

 Long-term patients (several years) with several appointments per year

 Long term patients (several years) with one appointment a year or less

1. Are 75% of your patients 65 or older?

 Yes

 No.

 I don't know

1. Here you have the opportunity to comment or add explanation to a question in writing

.................................................. .................................................. .................................................. .................................................. .................................................. .................................................. .................................................. ..................................................

References

1. A. Klein, Jared W.; Jackson, Sara L.; Osteer, Natalia V.; Peacock, Sue; Delbanco, Tom; Walker, Jan; Elmore, Joann G. Who Reads Their Doctor’s Notes? Examining the Association between Preconceptions and Accessing Online Clinical Notes. Journal of AHIMA 89; 2018;(1):28-33. Available from: <http://bok.ahima.org/doc?oid=302387>
2. Fossa AJ, Bell SK, DesRoches C. OpenNotes and shared decision making: A growing practice in clinical transparency and how it can support patient-centered care. J Am Med Informatics Assoc; 2018;(25):1153–9. PMID: 29982659
3. Gerard M, Chimowitz H, Fossa A, Bourgeois F, Fernandez L, Bell SK. The Importance of Visit Notes on Patient Portals for Engaging Less Educated or Nonwhite Patients: Survey Study. J Med Internet Res; 2018; (20):e191. doi: 10.2196/jmir.9196. PMID: 29793900
